# Solving a Hamiltonian Path Problem with a bacterial computer

**DOI:** 10.1186/1754-1611-3-11

**Published:** 2009-07-24

**Authors:** Jordan Baumgardner, Karen Acker, Oyinade Adefuye, Samuel Thomas Crowley, Will DeLoache, James O Dickson, Lane Heard, Andrew T Martens, Nickolaus Morton, Michelle Ritter, Amber Shoecraft, Jessica Treece, Matthew Unzicker, Amanda Valencia, Mike Waters, A Malcolm Campbell, Laurie J Heyer, Jeffrey L Poet, Todd T Eckdahl

**Affiliations:** 1 Department of Biology, Missouri Western State University, St Joseph, MO 64507, USA; 2 Department of Biology, Davidson College, Davidson, NC 28036, USA; 3 Department of Biology, North Carolina Central University, Durham, NC 27707, USA; 4 Department of Mathematics, Davidson College, Davidson, NC 28036, USA; 5 Department of Computer Science, Math and Physics, Missouri Western State University, St Joseph, MO 64507, USA; 6 Natural Science and Math Department, Johnson C. Smith University, Charlotte, NC 28216, USA

## Abstract

**Background:**

The Hamiltonian Path Problem asks whether there is a route in a directed graph from a beginning node to an ending node, visiting each node exactly once. The Hamiltonian Path Problem is NP complete, achieving surprising computational complexity with modest increases in size. This challenge has inspired researchers to broaden the definition of a computer. DNA computers have been developed that solve NP complete problems. Bacterial computers can be programmed by constructing genetic circuits to execute an algorithm that is responsive to the environment and whose result can be observed. Each bacterium can examine a solution to a mathematical problem and billions of them can explore billions of possible solutions. Bacterial computers can be automated, made responsive to selection, and reproduce themselves so that more processing capacity is applied to problems over time.

**Results:**

We programmed bacteria with a genetic circuit that enables them to evaluate all possible paths in a directed graph in order to find a Hamiltonian path. We encoded a three node directed graph as DNA segments that were autonomously shuffled randomly inside bacteria by a Hin/*hixC *recombination system we previously adapted from *Salmonella typhimurium *for use in *Escherichia coli*. We represented nodes in the graph as linked halves of two different genes encoding red or green fluorescent proteins. Bacterial populations displayed phenotypes that reflected random ordering of edges in the graph. Individual bacterial clones that found a Hamiltonian path reported their success by fluorescing both red and green, resulting in yellow colonies. We used DNA sequencing to verify that the yellow phenotype resulted from genotypes that represented Hamiltonian path solutions, demonstrating that our bacterial computer functioned as expected.

**Conclusion:**

We successfully designed, constructed, and tested a bacterial computer capable of finding a Hamiltonian path in a three node directed graph. This proof-of-concept experiment demonstrates that bacterial computing is a new way to address NP-complete problems using the inherent advantages of genetic systems. The results of our experiments also validate synthetic biology as a valuable approach to biological engineering. We designed and constructed basic parts, devices, and systems using synthetic biology principles of standardization and abstraction.

## Background

### Contemporary mathematical challenges to computation

**Figure 1:**
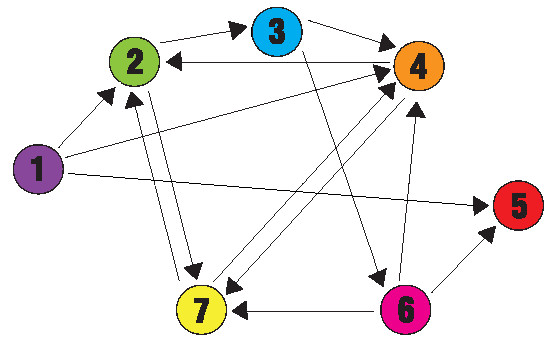
**A directed graph containing a unique Hamiltonian path**. The seven nodes are connected with fourteen directed edges. The Hamiltonian Path Problem is to start at node 1, end at node 5, and visit each node exactly once while following the available edges. Adleman programmed a DNA computer to find the unique Hamiltonian path in this graph (1→4→7→2→3→6→5).

Mathematicians and computer scientists alike are familiar with the computational complexity associated with problems referred to as NP-complete [1]. Such problems are included in a group of decision problems known as NP, or nondeterministic polynomial, which have solutions that, once found, can easily be shown to be correct. Although many NP problems can be solved quickly, NP-complete problems cannot, since their complexity grows combinatorially with linear increases in the problem size. These problems are significant because of their relationships to each other: every NP-complete problem can be cast in the form of any other using a polynomial-time algorithm, meaning that an efficient algorithm for one NP-complete problem can be used to solve all others. Expert computer programmers learn to recognize patterns in their codes that suggest a particular problem is NP-complete and, as a result, either settle for an approximate solution or abandon their attempt to obtain an exact one. The first problem proved to be NP-complete was the Boolean Satisfiability Problem (SAT), which is the problem of determining whether or not the variables in a logical expression can be assigned to make the expression true [2]. Other NP-complete problems include the Knapsack Problem, the Maximum Clique Problem, and the Pancake Problem. A version of the Pancake Problem, the Burnt Pancake Problem, was introduced in the only academic publication by Bill Gates [3]. The NP-complete problem addressed in this paper is the Hamiltonian Path Problem (HPP), in which a path must be found in a directed graph from a beginning node to an ending node, visiting each node exactly once. Figure 1 shows a directed graph with a unique Hamiltonian path from node 1 to node 5.

The serial approach that most silicon computer algorithms use is not well suited for solving NP-complete problems because the number of potential solutions that must be evaluated grows combinatorially with the size of the problem. For example, a Hamiltonian Path Problem for a directed graph on ten nodes may require as many as 10! = 3,628,800 directed paths to be evaluated. A static number of computer processors would require time proportional to this number to solve the problem. Doubling the number of nodes to 20 would increase the possible number of directed paths to 20! = 2.43 × 10^18^, increasing the computational time by 12 orders of magnitude. Improvement in computational capability could come from parallel processing and an increase in the number of processors working on a problem. Significant breakthroughs in this regard may be possible with the development of biological computing, because the number of processors grows through cell division.

### Biological computing

In a groundbreaking experiment, Leonard Adleman demonstrated an alternative to the serial processing of silicon computers by developing a DNA computer that could carry out parallel processing *in vitro *to solve the HPP in Figure 1[4]. The seminal work by Adleman inspired others to develop DNA computers capable of solving mathematical problems that are intractable to serial computing [5-7].

We asked whether it would be possible to move DNA computing inside bacteria that could function as a living computer with billions of processors. Programming bacteria to compute solutions to difficult problems could offer the same advantage of parallel processing that DNA computing brings, with the following additional desirable features: (1) bacterial systems are autonomous, eliminating the need for human intervention, (2) bacterial computers can adapt to changing conditions, evolving to meet the challenges of a problem, and (3) the exponential growth of bacteria continuously increases the number of processors working on a problem.

In a previous study, we reconstituted the *S. typhimurium *Hin/*hixC *recombinase system for use in *E. coli *[8]. In addition to its potential use in controlling the order and orientation of transgenes and for modeling syntenic genome relationships, the system has proved to be a useful tool in the development of bacterial computers. Recombination by Hin recombinase results in the inversion of DNA fragments that are flanked by a pair of *hixC *sites [9,10]. We demonstrated that Hin recombinase could invert either a single DNA fragment or multiple adjacent fragments in a single operation [8].

We used the Hin/*hixC *system to engineer living bacterial cells to calculate a solution to a variation of the Burnt Pancake Problem [8]. The problem involves sorting a set of burnt pancakes so that they all have the same orientation and are arranged in a particular order. Our biological representation of a burnt pancake was a functional DNA unit containing a promoter or a protein coding sequence, each flanked by a pair of *hixC *sites. We used the selectable phenotype of antibiotic resistance to identify bacteria that solved the BPP. Our results served as an important proof-of-concept that bacteria can function as parallel processors in the computation of solutions to a mathematical problem.

We sought to use our bacterial computing approach to solve a Hamiltonian Path Problem, as Adleman did with a DNA computer. With an appreciation for history, we designed a DNA-encoded version of Figure 1 to encode the HPP into DNA segments that could be inverted by Hin recombinase. To test the feasibility of solving the HPP *in *vivo, we designed, constructed, and tested bacteria that announced their arrival at a solution to a proof-of-concept three node HPP by producing colonies that fluoresced yellow.

## Results

### Genetic encoding of the Hamiltonian Path Problem

**Figure 2:**
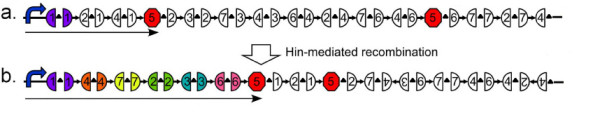
**Illustration of the use of split genes to encode a seven node Hamiltonian Path Problem**. a. The manner in which each of the directed edges in Figure 1 could be encoded in DNA is illustrated. The 5' half of each node gene is denoted by  and the 3' half is denoted by . DNA edges are depicted by gene halves connected by arrows and flanked by triangles that represent *hixC *sites. Transcription in the direction of the solid arrow would terminate early and result in the expression of only one marker gene. b. Hin-mediated recombination would randomly reshuffle the DNA edges into many configurations. One possible example of an HPP solution configuration with its marker gene halves reunited is illustrated. Transcription in the direction of the solid arrow would result in expression of the six marker gene phenotypes.

The design of our bacterial computer benefited from a series of abstractions of DNA sequence into the edges and nodes of a Hamiltonian path. The first abstraction treated DNA segments as edges of a directed graph. DNA edges flanked by *hixC *sites can be reshuffled by Hin recombinase, creating random orderings and orientations of edges of the graph. The second abstraction treated all nodes, except the terminal one, as genes split into two halves (Figure 2). The first (5') half of the gene for a given node is found on any DNA edge that terminates at the node, while the second (3') half of the gene is found on any DNA edge that originates at the node. The final abstraction was an arrangement of DNA edges that represented a HPP solution and exhibited a new phenotype. To place our proposed improvement of DNA computing in the historical context of the graph in Figure 1, we designed the constructs shown in Figure 2. Each node in the graph is represented by a gene that encodes an observable phenotype, such as antibiotic resistance or fluorescence. The exception to this is node 5, which is represented by a transcription terminator to ensure that it will be the last node in the Hamiltonian path. Each 5' half of a gene is denoted by the left half of a circle and each 3' half is denoted by the right half of a circle. Gene halves connected by arrows and flanked by triangular *hixC *sites are the flippable DNA edges. The order and orientation of the DNA edges determines the starting configuration, an example of which is illustrated in Figure 2a. Hin-mediated recombination of the 14 DNA edges could produce 1.42 × 10^15 ^possible configurations. Of these, a small fraction represent Hamiltonian paths with all of the node genes intact (see mathematical modeling section below for details). An example of one of these solution configurations is illustrated in Figure 2b. Bacterial colonies that contain an HPP solution will express a unique combination of phenotypes that can be detected directly or found by selection.

### Splitting GFP and RFP genes

Once we were convinced that our proposed *in vivo *DNA computer could solve a HPP, we chose a simpler three node graph for our first biological implementation of the problem. To execute our design, we needed to split two marker genes by inserting *hixC *sites. For each gene to be split, we had to find a site in the encoded protein where 13 specific amino acids could be inserted without destroying the function of the protein. We examined the three-dimensional structure of each protein candidate, chose a site for the insertion, built gene halves, and tested the reunited halves with the 13 amino acid insertion for protein function. We successfully inserted *hixC *sites into the coding sequences of both GFP and RFP without loss of fluorescence [11]. We inserted the *hixC *site between amino acids 157 and 158 in GFP, and between the structurally equivalent amino acids 154 and 155 in RFP. Each of the insertions extended a loop outside of the beta barrel structure of the fluorescent proteins. We also tested two hybrid constructs to ensure that they would not fluoresce. We assembled the 5' half of GFP with the 3' half of RFP and the hybrid protein did not fluoresce red or green (data not shown). Similarly, the 5' half of RFP placed upstream of the 3' half of GFP did not cause fluorescence (data not shown). In addition, none of the four half proteins fluoresced by themselves (data not shown). These results demonstrated the suitability of the GFP and RFP gene halves as parts for use in programming a bacterial computer to solve an HPP. Being able to split two genes enabled us to design a bacterial computer to solve an HPP for a three node directed graph.

### Mathematical modeling of bacterial computational capacity

**Figure 3:**
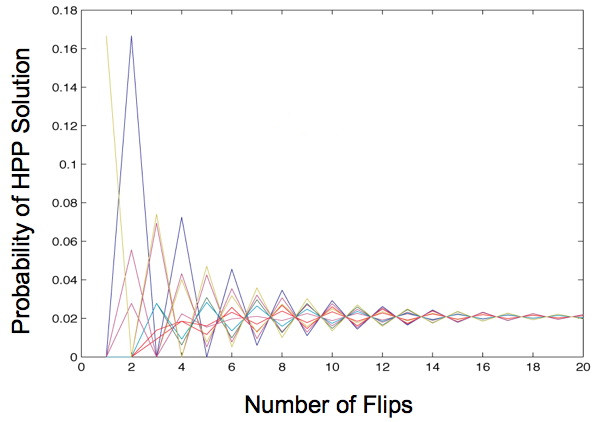
**Markov Chain model of solving a Hamiltonian Path Problem**. Each colored line represents a different starting configuration of a graph with four nodes and three edges. As the number of flips increases, the probability of finding a Hamiltonian path solution converges to 1/48, or about 0.02.

We used mathematical modeling to examine several important questions about the system. The first question is whether the order and orientation of the DNA edges in a starting construct affect the probability of detecting an HPP solution. During an HPP experiment, billions of bacteria cells will attempt to find a solution by random flipping of DNA edges catalyzed by Hin recombinase. We developed a Markov Chain model in MATLAB using the signed permutations of {1,2,...*n*} as the states of DNA edges in the HPP. We assumed that each possible reversal of adjacent DNA edges was equally likely. Using this transition matrix, we computed the probability that any starting configuration would be in any of the solved states after *k *flips. We conducted this analysis for a number of different graphs. Figure 3 shows one example of the results, for a graph with four nodes and three edges. The graph shows a relatively quick convergence to equilibrium, as was the case for all the graphs we analyzed. In this example, there are 48 possible configurations of the edges, only one of which is a solution. After about 20 flips, the probability that the edges are in the solution state (or any other state) is 1/48 (≈ 0.02). Consideration of the reaction rate reported for Hin recombinase [12] led us to conclude that equilibrium could be reached in the 3-node, 3-edge experiment that we intended to use as a proof-of-concept. Assuming that *E. coli *divides every 20–30 minutes and that we grow the cells for 16 hours, exceeding 20 flips should occur even if Hin recombinase catalyzes only one reaction per cell cycle.

We also used mathematical modeling to determine how many bacteria would be needed to have high confidence that, after Hin recombination, at least one cell would contain a plasmid with a true HPP solution. For the example of the graph in Figure 1, each HPP solution would have six DNA edges in the proper order and orientation followed by the remaining eight edges in any order and orientation. Because there are 8! ways to order the eight remaining edges, and two ways to orient each one, there are 8!·2^8 ^= 10,321,920 different configurations that are solutions, one example of which is shown in Figure 2b. There is a total of 14!·2^14 ^= 1.42 × 10^15 ^possible configurations of the edges (14! ways to order the edges, and two ways to order each one), many of which are not even valid connected paths in the graph, much less Hamiltonian paths. The probability of any one plasmid holding an HPP solution is *p *= (8!·2^8^)/(14!·2^14^). Assuming that the states of different plasmids are independent and that a sufficient number of flips has occurred to achieve a uniform distribution of the 14!·2^14^possible configurations, the probability that at least one of *m *plasmids holds an HPP solution is 1-(1-*p*)^m^. From this expression, we can solve for *m *to find the number of plasmids needed to reach the desired probability of finding at least one solution. For example, if we wanted to be 99.9% sure of finding an HPP solution, we would need at least one billion independent, identically distributed plasmids. A billion *E. coli *can grow overnight in a single culture. It should be noted, however, that it may take longer than that for Hin recombination to produce a uniform distribution of all possible plasmid configurations. Since each bacterium would have at least 100 copies of the plasmid, the computational capacity of a billion cells exceeds our needs by two orders of magnitude. Because the number of processors would be increasing exponentially, the time required for a biological computer to evaluate all 14!·2^14 ^configurations is a constant multiple of log(14!·2^14^), or approximately 14·log(14), while the time required for a conventional computer to evaluate the same number of paths would be a constant multiple of 14!·2^14^.

A key feature of our experimental design is the simplicity of detecting answers with phenotypes of red and green fluorescence resulting in yellow colonies. However, when our design is applied to a more complex problem such as the one presented in Figures 1 and 2, it is possible that a colony with a correct phenotype might have an incorrect genotype, resulting in a false positive. We considered the question of whether there are too many false positives to detect a true positive. Using MATLAB, we computed the number of true positives for the 14-edge graph in Figure 1 to be 10,321,920 and the number of total positives to be 168,006,848. The ratio of true positives to total positives is therefore approximately 0.06. Since all false positive solutions must have at least one more edge between the starting node and the ending node than in the true solution states, putative solutions could be screened using PCR. However, since the ratio of true to total positives gets smaller with the size of the problem, this approach becomes increasingly impractical. An alternative would be to conduct high throughput DNA sequencing of pooled putative solution plasmids.

Our mathematical modeling supported the conclusion that our experimental design could solve Hamiltonian Path Problems. As a proof-of-concept, we designed a simple directed graph with a unique Hamiltonian path and programmed a bacterial computer to find that path.

### Programming a bacterial computer

**Figure 4:**
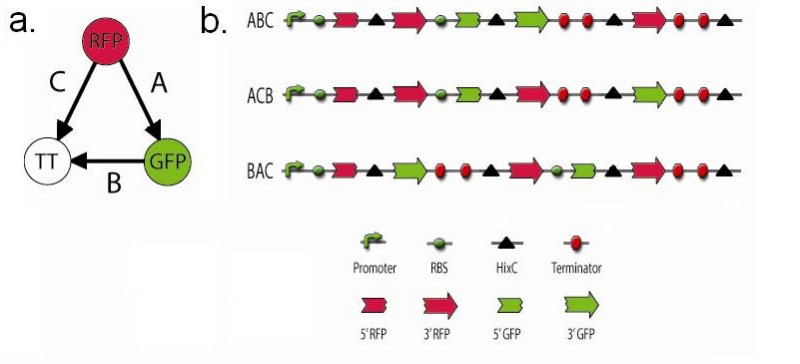
**DNA constructs that encode a three node Hamiltonian Path Problem**. a. The three node directed graph contains a Hamiltonian path starting at the RFP node, proceeding to the GFP node, and finishing at the TT node. b. Construct ABC represents a solution to the three node HPP. Its three *hixC*-flanked DNA segments are in the proper order and orientation for the GFP and RFP genes to be intact. ACB has the RFP gene intact but not the GFP gene, while BAC has neither gene intact.

Figure 4a shows the directed graph with three nodes and three edges that we chose to encode in our bacterial computer. The graph contains a unique Hamiltonian path starting at the RFP node, traveling via edge A to the GFP node, and using edge B to reach the ending TT node. Edge C, from RFP to TT, is a detractor. Figure 4b illustrates the DNA constructs we used to encode a solved HPP as a positive control and two unsolved starting configurations. Since the solution must originate at the RFP node and terminate at the GFP node, DNA edge A contained the 3' half of RFP followed by the 5' half of GFP. DNA edge B originated at GFP and terminated at TT, so its DNA segment has 3' GFP followed by the double transcription terminator. DNA edge C originated with the 3' half of RFP and terminated at TT. Each of the 5' gene halves included a ribosome binding site (RBS) upstream of its start codon in order to support translation.

**Figure 5:**
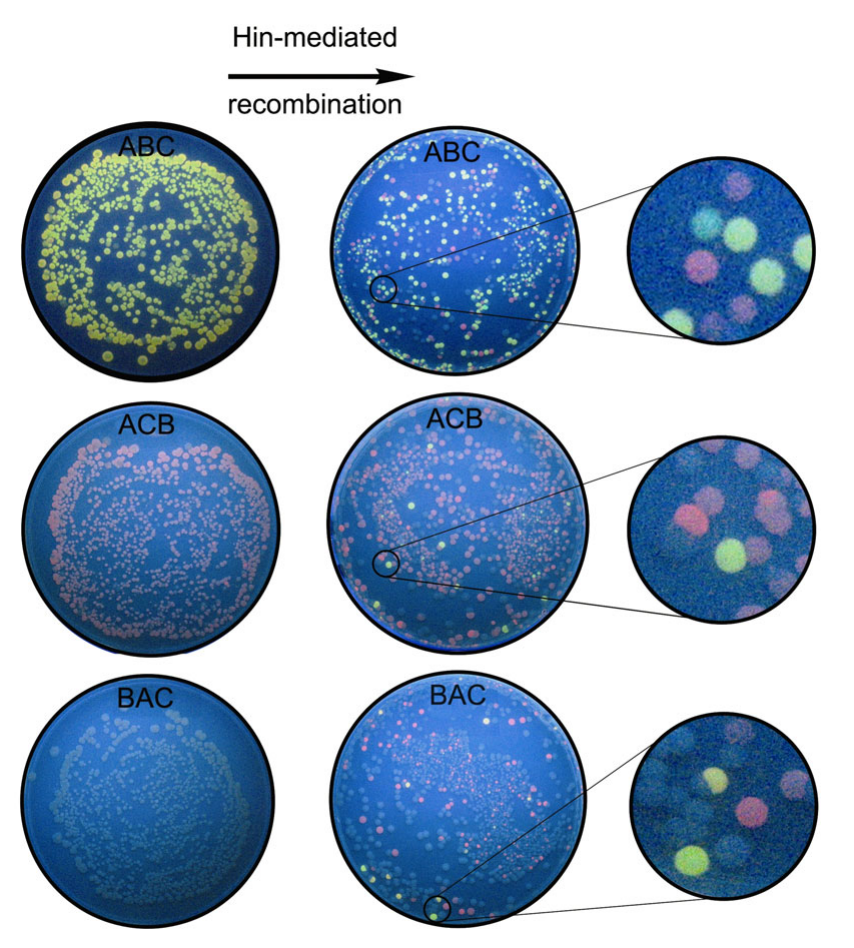
**Detecting solutions to a Hamiltonian Path Problem with bacterial computing**. Bacterial colonies containing each of the three starting constructs ABC, ACB, and BAC are shown on the left. Hin recombination resulted in the three plates of colonies on the right. The callouts include yellow colored colonies that contain solutions to the HPP.

As illustrated in Figure 4b, we designed an expression cassette to contain the three DNA edges. To ensure the solution begins at the RFP node, the cassette starts with a bacteriophage T7 RNA polymerase promoter, an RBS, and 5' RFP prior to the first *hixC *site. Construct ABC represents one of two HPP solutions since it begins with the RFP node, passes through GFP and ends with TT. Since both the RFP and GFP genes are intact, downstream of the promoter, in the correct orientation, and followed by the transcriptional terminators, ABC colonies should express both red and green fluorescence and appear yellow. A second solution is ABC', in which forward DNA edges A and B are followed by backwards DNA edge C. Bacteria containing this configuration are expected to fluoresce yellow, since RFP and GFP are intact and in forward orientation. Construct ACB has the RFP gene intact, in the correct orientation, and uninterrupted by transcriptional terminators, but its GFP gene halves are not united. As a result, this construct is predicted to produce red colonies. The BAC construct has neither RFP nor GFP intact and should not fluoresce at all. The three plates on the left side of Figure 5 show that all three constructs produced the predicted phenotypes in the absence of Hin recombinase: ABC colonies fluoresce yellow, ACB colonies fluoresce red, and BAC colonies show no fluorescence.

Random orderings of edges in the directed graph were produced by Hin-mediated recombination in a separate experiment using each of the three starting constructs ABC, BAC, or ACB. In a given experiment, bacteria were cotransformed with 1) a plasmid conferring ampicillin resistance and containing one of the three starting constructs and 2) a plasmid encoding tetracycline resistance with a Hin recombinase expression cassette. The resulting cotransformed colonies were grown overnight for isolation of plasmids containing the Hin-exposed HPP constructs. The isolated plasmids were then used in a second round of transformation into bacteria that expressed bacteriophage T7 RNA polymerase and plated on media containing only ampicillin (Figure 5). Ampicillin-resistant colonies were grown overnight to allow the T7 RNA polymerase to transcribe each plasmid in its final flipped state. Because each colony represented a single transformation event and Hin was no longer present, each colony contained isogenic plasmids and thus only one configuration of the three DNA edges. This experimental protocol was followed for each of the three starting constructs.

### Verifying bacterial computer solutions to a Hamiltonian Path Problem

Once Hin recombinase reorders the DNA edges of each of the constructs, a distribution of 48 possible configurations is expected. The positive control ABC construct should convert from its yellow fluorescent starting phenotype to the red and uncolored phenotypes of unsolved arrangements. The ABC recombination plate pictured in Figure 5 matched our prediction. We assumed that the double transcriptional terminator would function in reverse orientation, so that green colonies would not be possible in the experiment. However, green colonies on the ABC recombination plate indicate that TT did not block further transcription. The ABC recombination plate also shows a number of unusually colored colonies that were not expected, which we discuss later.

The ACB starting construct was expected to undergo Hin-mediated recombination to produce a variety of configurations, including a solution that requires at least two flips. Yellow fluorescent colonies representing putative HPP solutions are visible on the ACB recombination plate. The BAC starting configuration was three flips away from the nearest solution. Several examples of yellow fluorescent colonies on the BAC recombination plate are candidates for solutions to the HPP. As with the ABC recombination plate, we found unexpected colony colors on both the ACB and BAC recombination plates.

**Figure 6:**
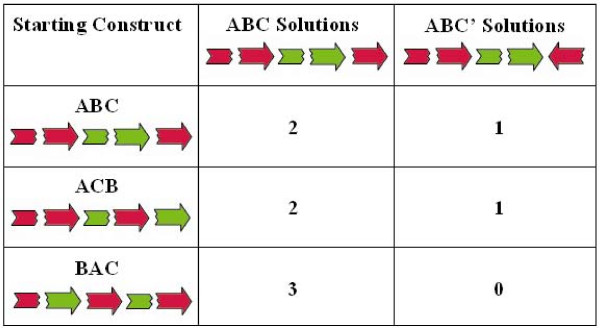
**DNA sequence verification of HPP solutions**. Three yellow fluorescent colonies from each of the three recombination plates were used for plasmid preparation and DNA sequencing. The number of ABC and ABC' solution genotypes found for each of the starting constructs is listed. The order and orientations of GFP (green) and RFP (red) gene halves for each of the starting constructs and solutions is illustrated.

Yellow fluorescent colonies on the ACB and BAC recombination plates provided preliminary evidence that the bacterial computer had solved both versions of the HPP. We wanted to verify this result by sequencing plasmid DNA to determine the genotypes of three yellow colonies from each of the ABC, ACB, and BAC recombination plates. All nine colonies had a genotype of ABC or ABC', in which the third DNA edge is in reverse orientation (Figure 6). Both of these configurations represent a solution to the HPP. These results verified that our bacterial computer had found true solutions to a three node HPP configured in two different starting orientations.

## Discussion

### Bacterial computer reveals novel phenotypes

We used the principles and practices of synthetic biology to design and build a bacterial computer that solved a Hamiltonian Path Problem. We successfully encoded a directed graph with three nodes and three edges into DNA and used Hin recombinase to rearrange the edges into a Hamiltonian path configuration that yielded a yellow fluorescent phenotype. We verified genotype solutions to the problem with DNA sequencing. Our engineered bacterial computer system functioned according to our expectations and solved the HPP unassisted by human intervention.

**Figure 7:**
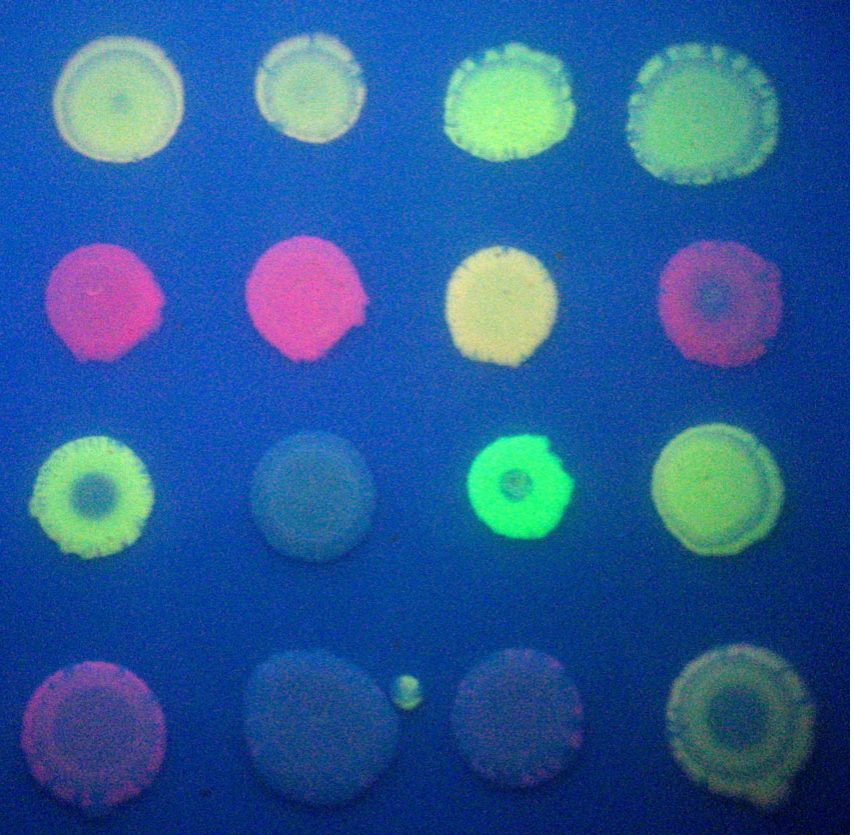
**Clones isolated from HPP recombination plates**. Selected colonies from ABC, ACB, and BAC recombination plates were grown overnight and replated. The results emphasize the diversity of colors produced by the bacterial computer in the HPP experiment.

Synthetic biology often reveals unexpected behaviors in engineered biological systems. We observed novel phenotypes produced by our bacterial computer that we had not predicted. We isolated bacteria with unexpected colors such as green, orange, pink, yellowish-green, and pale yellow (Figure 7). One possible explanation for these results is that some colonies may not be clonal. We replated colonies with unusual colors for colony isolation. Some colonies did exhibit more than one clone by producing colonies of more than one color. For the colonies of novel color that were truly clonal, promoterless transcription in the reverse direction could have produced low level gene expression [8,13]. For example, a construct that produced red color because of an intact RFP gene expressed by the T7 RNA polymerase promoter could have produced a low level of green with expression from intact GFP gene in the reverse orientation. Such a clone might appear to be orange in color. Another explanation for novel colors is mutation of the coding sequences for RFP and GFP, although we consider this to be less likely. Our system is behaving in unexpected ways in addition to its designed purpose of finding a solution to the HPP, which opens up new areas for investigation of Hin recombinase activity *in vivo*.

An iterative approach to synthetic biology is to examine a natural system, deconstruct it into component parts and devices, design and build an engineered system that performs new functions or tests hypotheses about the natural system, and evaluate the behavior of the engineered system. It should not be surprising that attempts to engineer biology produce results that are not easily explained without further research. But ignoring unexpected behavior would be a lost opportunity to advance our understanding of nature. Rather, the unpredictability of engineered biological systems should return synthetic biologists to another iteration of examination, deconstruction, design, and testing. The unexplained behavior of our system was a good example of the dual benefits of synthetic biology. In addition to engineering a bacterial computer to solve the HPP, our work provided unanticipated opportunities for further investigation of the mechanism by which Hin recombinase functions *in vivo *and the means by which a complex population of plasmids is maintained in our bacterial computer.

### Hin-mediated recombination non-equilibrium

A test of whether or not Hin recombinase has achieved equilibrium in our experiments is to compare the predicted and observed frequencies of colony phenotypes. For the three node directed graph, there are 3!·2^3 ^= 48 possible configurations of the three DNA edges. At equilibrium, each of these is expected to occur at a frequency of 1/48. As a result of observing green fluorescent colonies, we will assume for the purpose of this analysis that the double terminator did not function in reverse orientation in our experiments. With this assumption, only the configuration C'AB results in green fluorescence, so colonies with this phenotype are expected at a rate of 1/48, or about 2%. However, green colonies appear at less than this rate in all the experiments. Yellow fluorescence can be produced only by the two configurations ABC and ABC', yielding a rate of 2/48, or about 4%. However, yellow fluorescent colonies predominate in the ABC experiment and are less common than 4% in ACB and BAC experiments. Red fluorescence requires either a configuration with C in the first position or one with A in the first position but not forward B in the second position. There are 14 configurations that satisfy these criteria so the expected frequency of red fluorescent colonies is 14/48, or about 29%. However, red fluorescence is the dominant color on ACB plates and is rare on the ABC and BAC plates. Configurations with A', B, or B' in the first position or with C' followed by any combination except AB will yield no fluorescence. There are 31 configurations that meet these criteria, so the expected frequency is 31/48, or about 65%. However, uncolored colonies dominate the BAC plate and do not approach this expected rate on ABC and ACB plates. Overall, these results show that each experiment retained a greater frequency of original colony color than was predicted at equilibrium. This supports the conclusion that Hin recombinase had not reached equilibrium. These results are in agreement with the conclusion of our previous study that Hin recombinase flipping had not reached equilibrium after 11 hours, perhaps because we chose to omit the Recombination Enhancer element [8].

We have considered possible explanations for the observed Hin-mediated recombination non-equilibrium. Lim *et al. *reported that Hin recombinase requires negative supercoiling in its substrate plasmid, and that recombination removes two negative supercoils during a reaction [14]. The supercoiling density has been reported to be 8–12 supercoils per plasmid [15], and if new supercoils were not introduced until DNA replication, then perhaps Hin recombinase can perform only 4–6 reactions with each plasmid per generation. Although our mathematical modeling revealed that equilibrium was achieved in 20 reactions, perhaps replication of plasmids early in the experiment increased the frequency of starting configurations to levels that could not be achieved by configurations that require more recombination reactions. In other words, the starting configurations might produce a type of founder effect that was still visible on the final recombination plates and not accounted for in our mathematical model.

### Scaling Hamiltonian Path Problems

We considered the question of what would be required for our bacterial computer to find the Hamiltonian path in directed graphs of increasing size. In addition to listing the GFP and RFP genes used to solve the three node directed graph, Table 1 lists specific proposals for split genes that could be used for directed graphs containing 4–7 nodes. Each of the genes chosen produces a phenotype that could be observed in the presence of the other phenotypes. In addition to the GFP and RFP genes used in the current study, β-galactosidase is proposed for its ability to produce blue colonies and three antibiotic resistance genes not used in the experimental protocol are proposed. The graph in Figure 1 could be addressed if we were able to insert a *hixC *site into the four additional genes without disrupting the functions of the encoded proteins and in such a way that a hybrid of halves of any two genes did not replicate any of the six phenotypes. If we could split the four additional genes, then we could program our bacterial computer to solve the same HPP *in vivo *that Adelman solved *in vitro *with a DNA computer. As indicated in Table 1, our approach could be used to find the Hamiltonian path in a directed graph containing N nodes by using N-1 split genes. Notably, the effort required to split genes increases linearly although the complexity of the problem increases combinatorially.

**Table 1:** Proposed split genes for solving increasingly larger Hamiltonian Path Problems

**Directed Graph**	**Split Genes**
**3 nodes**	GFP, RFP
**4 nodes**	GFP, RFP, β-Gal
**5 nodes**	GFP, RFP, β-Gal, Chl
**6 nodes**	GFP, RFP, β-Gal, Chl, Kan
**7 nodes**	GFP, RFP, β-Gal, Chl, Kan, Eryth
**N nodes**	N-1 Split Genes

Split genes that would be needed to program a bacterial computer to find the Hamiltonian path in a directed graph with the number of nodes indicated are listed. The 3 node proposed was successfully implemented in the current study. GFP = green fluorescent protein gene, RFP = red fluorescent protein gene, β-Gal = β-galactosidase gene, Chl = chloramphenicol resistance gene, Kan = kanamycin resistance gene, Eryth = erythromycin resistance gene.

As described earlier, one in every 138,378,240 of the possible configurations of the edges of the graph in Figure 1 is a Hamiltonian path. Since this is roughly the number of plasmids in a typical experiment, finding a solution would require a more efficient screening mechanism. We could increase the probability of finding a true HPP solution if we enhanced Hin recombinase function by adding the Recombination Enhancer [16] or if antibiotic selection were used at time points prior to the end of the experiment. If even larger graphs were to be addressed, selection for partial solutions would be necessary and the problem might have to be divided into stages. For example, bacteria that had successfully solved the first half of the graph could be assigned a higher fitness than those that had failed to reach this milestone. In this way, directed evolution could be used to guide the population of bacterial processors toward a final solution.

## Conclusion

The manner in which the complexity of NP-complete problems such as the HPP grows is combinatorial with respect to linear increases in their size. This makes finding solutions to such problems a formidable challenge to computation. The success of our experiments to program a bacterial computer to solve a three node HPP represents an important step in the development of bacterial computers that can address this challenge. We have established that bacterial computers can function as a culture of exponentially growing cells that can evaluate an exponentially increasing number of solutions to an NP complete mathematical problem and determine which of them is correct.

The successful design and construction of a system that enables bacterial computing also validates the experimental approach inherent in synthetic biology. We used new and existing modular parts from the Registry of Standard Biological Parts [17] and connected them using a standard assembly method [18]. We used the principle of abstraction to manage the complexity of our designs and to simplify our thinking about the parts, devices, and systems of our project. The HPP bacterial computer builds upon our previous work and upon the work of others in synthetic biology [19-21]. Perhaps the most impressive aspect of this work was that undergraduates conducted every aspect of the design, modeling, construction, testing, and data analysis.

## Methods

### Construction of HPP parts and devices

Materials used in molecular cloning procedures were as follows. Plasmid preparations were conducted using either the Zippy Plasmid Miniprep Kit from Zymo Research or the QIAprep Spin Miniprep Kit from Qiagen. Gel fragment purifications were performed with either the Zymo Research Zymoclean DNA Recovery Kit or the Qiagen QiaExII polyacrylamide gel purification kit. Competent *E. coli *JM109 or T7 Express *I*^*q *^competent cells were purchased from New England Biolabs. Transformants were plated on LB media or grown in LB broth containing 100 ug/ml amplicillin, or 50 ug/ml tetracycline, or both. Polyacrylamide gel electrophoresis was conducted using 7% or 12% acrylamide in TBE buffer and agarose gel percentages ranged from 1% to 3% agarose in TAE buffer.

We designed and built all the basic parts used in our experiments as BioBrick compatible parts and submitted them to the Registry of Standard Biological Parts [17]. Key basic parts and their Registry numbers are: 5' RFP (BBa_I715022), 3' RFP (BBa_ I715023), 5' GFP (BBa_I715019), and 3' GFP (BBa_I715020). All basic parts were DNA sequence verified. The basic parts *hixC *(BBa_J44000), Hin LVA (BBa_J31001) were used from our previous experiments [8]. The parts were assembled by the BioBrick standard assembly method [18] yielding intermediates and devices that were also submitted to the Registry. Important intermediate and devices constructed are: Edge A (BBa_S03755), Edge B (BBa_S03783), Edge C (BBa_S03784), ABC HPP construct (BBa_I715042), ACB HPP construct (BBa_I715043), and BAC HPP construct (BBa_I715044). We previously built the Hin-LVA expression cassette (BBa_S03536) [8].

After construction of the A, B, and C DNA edges, DNA sequencing was performed to verify that they were correct. These intermediates were combined to produce the three HPP constructs ABC, ACB and BAC, which were also sequence verified. The HPP constructs were then cloned downstream of the bacteriophage T7 RNA polymerase promoter, an RBS element, and the 5' half of RFP. The Hin recombination expression cassette was used as previously constructed [8]. It included the lactose promoter, RBS, the coding sequence for Hin recombinase with a LVA degradation tag, and a double transcription terminator. The cassette was cloned into plasmid pSB3T5, which contains a tetracycline resistance gene and an origin of replication that allowed it to be maintained alongside the replication origin of the pSB1A3 plasmids used for the HPP constructs.

### Splitting genes

In order to split genes by insertion of *hixC *sites, we developed an online tool for primer design [22]. The software requires input of the coding sequence for the gene to be split and the point in the sequence where it is to occur. Since the *hixC *site is 26 bp and the BioBrick scar is 6 bp on each side of it, the insert needed to be 38 bp. This is not a multiple of 3 and therefore disrupts the reading frame after the insertion. Since choosing the 39th base will result in either glutamate or aspartate and can slightly modify the melting point of the primers, the software allows this choice to be made. The output is a PCR primer pair for the 5' and 3' gene halves. We used this tool to generate primers for the GFP and RFP genes that we wished to split. The resulting primers were used in PCR with cloned GFP and RFP genes as templates. The resulting DNA was cloned into the plasmid vector pSB1A3 and used for transformation. Putative clones were sequenced in order to choose clones with no mutations.

### Hin-mediated recombination of HPP constructs

ABC, ACB, and BAC starting constructs were used to transform T7 Express *I*^*q *^competent cells. These cells express the bacteriophage T7 RNA polymerase needed for expression of the HPP node genes. The transformants were plated on LB with ampicillin. After overnight incubation at 37°C, the plates were allowed to incubate at room temperature for an additional two days in order for fluorescence to develop. Pictures of these control plates were then taken for use in Figure 5.

Exposure of ABC, ACB, and BAC starting configurations to Hin recombinase was accomplished by cotransformation of JM109 cells with pSB1A3 plasmids containing the three constructs and a pSB3T5 plasmid containing the Hin expression cassette. The cotransformants were plated onto LB agar with ampicillin and tetracycline. Colonies were then pooled and grown in LB media overnight. Plasmid DNA was purified from each of the three recombination cultures and used to transform T7Express *I*^*q *^competent cells. The transformants were plated on LB agar with ampicillin only so that the Hin expression plasmid would be lost and no further recombination would occur. The resulting plates were photodocumented for use in Figure 5.

### Verification of HPP solutions by DNA sequencing

Selected colonies from the ABC, ACB, and BAC recombination plates were used for plasmid preparations. The plasmids were subjected to DNA sequencing using three primers. Primer RFP1 has the sequence 5' CGGAAGGTTTCAAATGGGAACGTG 3' and binds to the 5' RFP gene fragment that precedes each of the HPP constructs. Primer GFP2 has the sequence 5' TACCTGTCCACACAATCTGCCCTT 3' and binds to the 3' GFP coding sequence, which can occur in any of the three positions or in either orientation in a given HPP clone. Finally, we used primer G00101 (5' ATTACCGCCTTTGAGTGAGC 3'), which binds in reverse orientation to plasmid DNA downstream of the HPP constructs. All sequencing reactions were performed by the Clemson University Genomics Institute.

## Competing interests

The authors declare that they have no competing interests.

## Authors' contributions

JB, KA, OA, STC, WD, LH, ATM, NM, JT, MU, AV, MW, AMC, and TTE designed, constructed, confirmed and submitted project parts to the Registry of Standard Biological Parts, built and tested constructs to solve the HPP pathway using a bacterial computer, and verified HPP solutions. JOD, MR, AS, LJH, and JLP conducted mathematical modeling of the HPP. JB, AMC, LJH, JLP, and TTE wrote the manuscript. All authors read and approved the final manuscript.
